# ABCD approach at the #7119 center, telephone triage system in Tokyo, Japan; a retrospective cohort study

**DOI:** 10.1186/s12873-022-00625-5

**Published:** 2022-04-19

**Authors:** Atsushi Sakurai, Sachiko Ohta, Jun Oda, Takashi Muguruma, Takeru Abe, Naoto Morimura

**Affiliations:** 1 Emergency Telephone Consultation Centre, Tokyo Medical Association, 2-5 Kandasurugadai, Chiyoda-ku, Tokyo, 101-8328 Japan; 2 Division of Emergency and Critical Care Medicine, Department of Acute Medicine, Nihon University School of Medicine, Oyaguchikamichou 30-1, Itabashi-ku, Tokyo, 173-8610 Japan; 3 Department of Pharmaceutical and Medical Business Sciences, Nihon Pharmaceutical University, 3-15-9 Yushima, Bunkyo-ku, Tokyo, 113-0034 Japan; 4 Research and Analysis, Center for Health Service Outcome Research and Development, 23-17-408 Sakuragaokashou, Shibuya-ku, Tokyo, 150-0031 Japan; 5 Department of Traumatology and Acute Critical Medicine, Graduate School of Medicine, Osaka University, 2-2 Yamadaoka, Suita, 565-0871 Japan; 6 Department of Emergency Medicine, Yokohama City University Graduate School of Medicine, 3-9 Kanazawa-ku Fukuura, Yokoyama-city, Kanagawa, 236-0004 Japan; 7 Advanced Critical Care and Emergency Center, Yokohama City University Medical Center, 4-57 Minami-ku Urafunemachi, Yokoyama city, Kanagawa, 232-0024 Japan; 8 Department of Emeregency Medicine, Teikyo Univeristy School of Medicine, 2-11-1 Kaga, Itabashi-ku, Tokyo, 173-8606 Japan

**Keywords:** Telephone triage, ABCD approach, Dispatch

## Abstract

**Background:**

The algorithm and protocol of the #7119 telephone triage in Tokyo, Japan, had been originally established and consists of three steps. In this study, we investigated the outcome of patients treated with physiological abnormality (ABCD approach: A, airway; B, breathing; C, circulation, and D, dysfunction of central nervous system) in step 2 during the #7119 telephone triage and clarified the meaning of evaluation of this approach.

**Methods:**

We retrospectively reviewed data from the Tokyo Fire Department from January 2016 to December 2017. Almost all the patients triaged using the ABCD approach were transferred to the hospital by ambulance and assigned severity by a physician. We divided patients into groups with combinations of 15 patterns including A, B, C, D, AB, AC, AD, BC, BD, CD, ABC, ABD, ACD, BCD, and ABCD. We compared the proportion of severe cases in each group using a Fisher's exact test, followed by residual analysis.

**Results:**

We analyzed 13,793 cases triaged using the ABCD approach. In this analysis, 31% of total cases were assessed as severe cases. Groupwise analysis showed that the proportion of severe cases was significantly higher in the AD, BC, CD, ABD, and ABCD groups, while it was significantly less in the C and AB groups than in the total cases.

**Conclusion:**

At the #7119 telephone triage, we can pick up the severe cases by the ABCD approach. This may contribute to the prompt transportation of severe patients to hospitals by dispatching ambulance cars using the #7119 telephone triage methods.

## Introduction

Telephone triage services have been established in several countries [1–4] and play an important role in the management of ambulance use and adjustment of primary care workload [5, 6]. The Tokyo metropolitan government has also established a telephone consultation center (the #7119 center), which has been providing a 24-h a day and 7-days a week service since 2007. It operates a nurse-run telephone advice line that aims to refer callers to the most appropriate services or to provide them with advice about how to care for their condition. Each consultation is classified into one of five triage categories (red, immediately call 119 for an ambulance; orange, urgently seek help within approximately 1 h; yellow, urgently seek help within approximately 6 h; green, non-urgent case with need to seek help within the next 24 h and blue, non-urgent case with no need to attend hospital or clinic) based on perceived severity [6, 7].

These services of #7119 are expected to contribute to efficient, clinically appropriate health care, and to avoid delays in the provision of emergency care in life-threatening cases. With this point of view, we established the new original algorithm and protocol for telephone triaging at the #7119 center by an expert-based decision of several emergency physicians [6, 7], referring to the Manchester Triage System (MTS) [8] and the telephone triage protocol for nurses at Portland, Oregon [9]. The #7119 protocol is also computerized and uses a symptom-based triage support system, making it a kind of computer decision support systems.

The flow chart of the triage algorithm in #7119 has been originally established by us and consists of three steps. Details of these steps have been described previously [7]. In brief in Step 1, a call handler (nurse) receives a patient’s call and collects information about the patient’s identification and reason for calling. If certain key words occur—especially cardiac arrest, no respiration, no pulse, submersion, or cold body—the call handler immediately connects the call to the emergency center to dispatch an ambulance. At a next phase of consultation with Step 2, we established the system of telephone triage using the ABCD approach: airway (A), breathing (B), circulation (C), and dysfunction of the central nervous system (D). In Step 2, the telephone consultation nurse asks the patient questions regarding the presence or absence of severe, abnormal physiological signs; this is similar to the ABCDE approach of Advanced Trauma Life Support (ATLS) [10] or first order (vital sign) modifiers in Canadian Emergency Department Triage and Acuity Scale (CTAS) [11]. The formulation of the mnemonic ABC has its roots in cardiopulmonary resuscitation established by Safar and Kouwenhoven in the 1950s [12, 13]. The ABCDE approach has subsequently been developed for application in all clinical emergencies for immediate assessment and treatment [14]. Olgers et al. reported the effectiveness of the ABCDE approach in the emergency department in potentially medically ill patients [15]. In the MTS factor, A, B, C, and D were picked up as discriminators of life threating factors [8]. In Step 3, there are 98 symptom-specific protocols for injuries or disease, including 18 for pediatric cases. Triage nurses ask patients in order from step 1 to step 3, and stop triage if cases match step 1 or 2, and immediately connects the call to the emergency center to dispatch an ambulance. These cases finished the triage process in steps 1 or 2. Details of the process at #7119 have been previously reported [7].

Several validated emergency scales dedicated to triage patients at emergency department (ED) admission exist like CTAS, Emergency Severity Index (ESI), [16] and MTS [8]. Many validation studies have been performed in the ED in the past [17–20]. However, validation studies on telephone triage protocol for physiological sign evaluation like the ABCDE survey have not been reported to date. Fortunately, we had access to the entire data of ambulance cases in Tokyo as almost all ambulance dispatches transfer patients to hospitals. Furthermore, we could estimate the outcome of almost all cases triaged using Step 2 because these cases are allocated to emergent category, and public ambulances then transport these patients to hospitals and record their outcome. Accordingly, the aim of this study was to investigate the outcome of cases triaged using ABCD approach, and to clarify the meanings of evaluation of physiological abnormality at a telephone triage.

## Methods

This is a retrospective cohort study. We retrospectively reviewed the data of the Tokyo Fire Department (TFD) from January 1, 2016 to December 31, 2017. Nurses triaged 152,145 cases in 2016 and 172,551 cases in 2017.

### Severity of patients at hospitals

In this study, the severity of a patient’s condition on emergency admission at a hospital was classified into one of five categories—dead, lethal, severe, moderate, and mild. In Japan, almost all patients who are transferred to hospital by ambulance car are assigned a severity grade by a physician upon arrival at the hospital, and these are recorded and aggregated publicly by the ambulance staff. This system was established by the Fire and Disaster Management Agency in Japan in 2006, and the definition of each severity category is shown in Table 1 [21]. This administrative guideline enabled us to acquire data on patients’ outcome of severity for all patients transferred to the hospital by ambulance. The severity was determined by the ER physician upon arrival of the ambulance at the hospital. In this study, the outcome of cases with severity was defined as moderate, severe, lethal, and dead (Table 1), and these patients may be admitted to the hospital or died.

**Table 1:** Definition of the severity categories of ambulance delivery cases in Japan

Severity	Definition
Mild	Not admitted
Moderate	Admitted without a life-threating condition
Severe	Admitted with possibility of a life-threating condition^a^
Lethal	Admitted with a life-threating condition^b^
Dead	Confirmed death

^a^Possibility of a life-threating condition means patients with a life-threating condition, but not lethal or dead

^b^Life-threating condition means the following: a) patients with a risk of cardiac or respiratory arrest; b) patients who have undergone cardiopulmonary resuscitation

### Method with ABCD approach by triage nurses

Triage nurses of #7119 are registered nurses and most of them have clinical experiences prior to employment in TFD. In TFD, triage nurses undergo off-the-job training with lectures and case studies for 2 weeks and on-the-job training for 1 month. Triage nurses asked questions based on the ABCD approach regarding severe, abnormal physiological signs related to A, B, C, and D. In this regard, a nurse may use the following questions over the telephone to enquire about abnormal physiological signs: A) “Can you speak normally?”, B)”Do you have short of breath?”, C)”How about your complexion. Is it pale? Do you have cold sweating?”, D) “Do you respond normally?” or”Does he or she respond normally?” Triage nurses ask all questions consecutively from A to D despite any physiological abnormal sign. If the consultation nurse feels there is a severe physiological abnormality, as reflected from the answers, he or she must assign the category red of triage level (most emergent level) and must connect to the 119 center to activate the emergency ambulance system.

### Data with ABCD approach (Step 2)

Data collected in ABCD approach were age; gender; questions regarding severe abnormal physiological signs relating to airway (A), breathing (B), circulation (C), and dysfunction of the central nervous system (D), and whether they led to the transportation of the patient to the hospital and outcome of patients’ severity at the hospital. As abnormal physiological signs in one case can exist from only 1 to 4, combinations of these signs yielded 15 patterns including A, B, C, D, AB, AC, AD, BC, BD, CD, ABC, ABD, ACD, BCD, and ABCD. The proportion of cases with severity was calculated using the formula: (number of moderate, severe, lethal, and dead cases) / (total number of cases).

#### Statistical analyses

Based on severity grouping, we first compared the outcomes of each group. The Fisher's exact test, followed by residual analysis, was conducted to examine the possibility of differences in outcomes among the 15 classified groups. Second, regarding the ABCD factors, we established interaction terms from each single item of four, ABCD, approaches all possible interactions with two to four combinations. In addition, a binary outcome was defined as the presence or absence of severity. To identify an ABCD factor, a multiple logistic regression analysis with stepwise variable elimination was then conducted using age, sex, each ABCD factor, and their interaction terms as independent variables. Regarding ABCD factor, we included each variable as an independent factor in a regression model, as well as all possible interaction terms, e.g. A, B, C, D, A*B, A*C, A*D, B*C, B*D, C*D, A*B*C, A*B*D, A*C*D, B*C*D, and A*B*C*D. We set *p* < 0.20 to include a variable into the model and *p* > 0.10 to exclude a variable from the model. Statistical significance was set at *P* < 0.05.

#### Ethics

This study was approved by the Ethics committee of the Nihon University Hospital (protocol number: 20220110). All data are anonymized, no patient identifiable data were recorded at any time, hence there was no need for informed consent from participants. Ethics committee of the Nihon University Hospital approved informed consent waiver. The research was conducted in accordance with the Declaration of Helsinki.

## Results

**Fig. 1:**
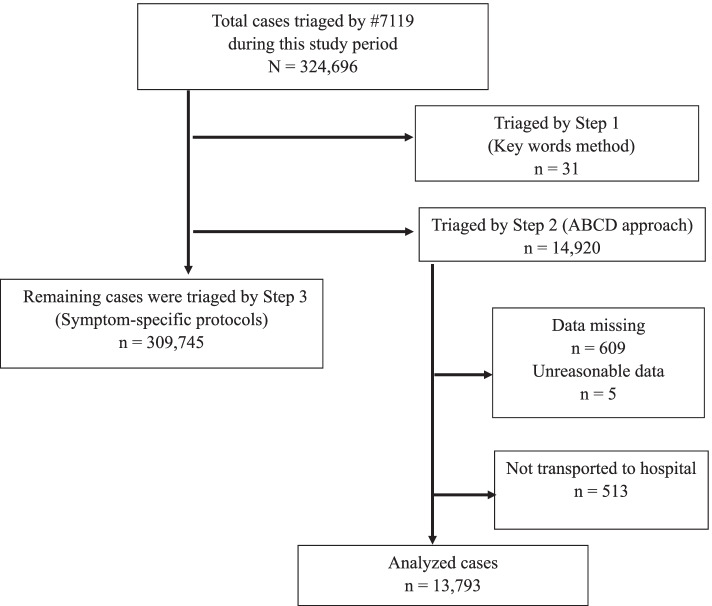
Overview of patient flow

Figure 1 shows an overview of patient flow, depending on each step of the #7119 telephone triage. During this study’s period, 324,696 cases were triaged by nurses. Our study included 14,920 (5%) cases triaged using ABCD approach. In ABCD approach, there were 609 cases with missing data (age, gender, outcome) and 5 cases with unreasonable cases, in which all physiological signs of A, B, C, and D were not checked and 513 cases were not transported because the patient refused to be transported to the hospital, and the outcome was unknown. We excluded these cases because we could not obtain information on severity. We consequently analyzed the data for 13,793 patients who were transported to the hospital through an ambulance dispatch.

The results of the analysis of ABCD approach triaging are shown in Tables 2, 3 and 4 (The result of this part is divided to three parts of each table). The outcome in 31% of total cases was cases with severity; either moderate, severe, lethal, or dead. There was no case in the BD group. Age and gender were not deviated at each group. The proportion of cases with severity was significantly higher in the AD (53%), BC (34%), CD (35%), ABD (58%), and ABCD (52%) groups, whereas it was significantly lower in the C (30%) and AB (22%) groups, as compared to that of the total cases (31%) as per the residual analysis. Factor D (D group with the combination of other groups) had a significant relationship with cases with severity.

**Table 2:** Summary of cases in every group triaged using ABCD approach

Group	A	B	C	D
Total number	50	7	8410	4
Age (median)	0–89(16)	1–74(40)	0–104(41)	1–82(13)
Gender (male: %)	24(48)	4(57.2)	3900(46.4)	3(75)
Outcome (%)				
Mild	37 (71.2)	5 (62.5)	5,905 (68.1)	4 (100)
Moderate	12 (23.1)	1 (12.5)	2,393 (27.6)	0 (0)
Severe	1 (1.9)	1 (12.5)	89 (1.0)	0 (0)
Lethal	0 (0)	0 (0)	22 (0.3)	0 (0)
Dead	0 (0)	0 (0)	1 (0)	0 (0)
The cases of severity: proportion (%)^a^	13 (26)	2 (29)	2505 (30)	0 (0)
Expected value	16	2	2668	1
Standardized residual	0.7	0.1	3.1	1.1
P value of residual analysis	0.384	0.585	< 0.001	0.173

^a^The cases of severity were defined as moderate, severe, lethal and dead, and the proportion of the cases of severity was calculated using the formula: (amount from moderate, severe, lethal and dead ceases) / (amount of total cases)

**Table 3:** Summary of cases in every group triaged using ABCD approach

Group	AB	AC	AD	BC	BD	CD
Total number	134	7	96	3795	0	791
Age (median)	0–96(8)	0–92(6)	0–101(45.5)	0–102(39)	-	0–100(17)
Gender (male: %)	63(47.1)	3(42.9)	53(55.3)	1656(43.7)	-	407(51.5)
Outcome (%)						
Mild	105 (75.0)	6 (86)	45 (41)	2,504 (64)	-	514(61)
Moderate	24 (17.1)	1 (14)	36 (33)	1185 (30)	-	228 (27)
Severe	4 (2.9)	0 (0)	11 (10.)	87 (2.2)	-	28 (3.3)
Lethal	1 (0.7)	0 (0)	4 (3.7)	19 (0.5)	-	17 (2.0)
Dead	0 (0)	0 (0)	0 (0)	0 (0)	-	4 (0.5)
The cases of severity (%)^a^	29 (22)	1 (14)	51 (53)	1291 (34)	-	277 (35)
Expected value	43	2	30	1204		251
Standardized residual	2.1	0.8	-3.7	-2.5	-	-1.6
P value of residual analysis	0.012	0.322	< 0.001	< 0.001	-	0.040

^a^The cases of severity were defined as moderate, severe, lethal and dead, and the proportion of the cases of severity was calculated using the formula: (amount from moderate, severe, lethal and dead ceases) / (amount of total cases)

**Table 4:** Summary of cases in every group triaged using ABCD approach

Group	ABC	ABD	ACD	BCD	ABCD	Total
Total number	36	48	37	345	33	13,793
Age (median)	1–92(32)	0–99(51)	0–83(29)	0–104(24)	0–93(5)	0–104(39)
Gender (male: %)	18(50)	28(58.4)	21(56.8)	157(45.6)	17(51.6)	6354(46.1)
Outcome (%)						
Mild	26 (68)	20 (40)	21 (51)	210 (57)	16 (47)	9,418 (66)
Moderate	7 (18)	22 (44)	10 (24)	109 (30)	10 (29)	4,038 (28)
Severe	2 (5.3)	3 (6.0)	4 (9.8)	16 (4.4)	4 (12)	250 (1.7)
Lethal	1 (2.6)	3 (6.0)	1 (2.4)	9 (2.5)	2 (5.9)	79 (0.6)
Dead	0 (0)	0 (0)	1 (2.4)	1 (0.3)	1 (2.9)	8 (0.1)
The cases with severity: proportion (%)^a^	10 (28)	28 (58)	16 (43)	135 (39)	17 (52)	4375 (32)
Expected value	11	15	12	109	10	-
Standardized residual	0.4	-3.3	-1.2	-2.4	-2.0	-
P value of residual analysis	0.611	< 0.001	0.131	0.003	0.014	-

^a^The cases with severity were defined as moderate, severe, lethal and dead those, and the proportion of the cases with severity was calculated using the formula: (amount from moderate, severe, lethal and dead ceases) / (amount of total cases)

**Table 5:** Severity and associated factors of ABCD approach by the telephone triage

Variants	B	OR	95% C.I. for OR		*p* value
			Lower	Upper	
Age	0.025	1.025	1.023	1.027	< 0.0001
Gender (Male)	0.104	1.234	1.142	1.349	< 0.0001
Factor B	0.110	1.245	1.150	1.349	< 0.0001
Factor D	0.290	1.786	1.574	2.027	< 0.0001

Only significant variables were presented in the table, after the stepwise variable elimination. The model included each variable as an independent factor in a regression model, as well as the interaction terms, e.g. A, B, C, D, A*B, A*C, A*D, B*C, B*D, C*D, A*B*C, A*B*D, A*C*D, B*C*D, and A*B*C*D

*OR* Odds Ratio, *C.I*. Confidence interval

Binary logistic regression analysis was conducted based on age, sex, each ABCD factor, and all possible interaction terms of ABCD factors. Age (OR, 1.025; 95% CI, 1.023–1.027), male sex (OR, 1.234; 95% CI, 1.142–1.1349), and factor B (OR, 1.245; 95% CI, 1.150–1.349) and factor D (OR, 1.786; 95% CI, 1.574–2.027) were significantly (*p* < 0.0001) associated with cases with severity (Table 5: Only significant variables were presented in the table, after the stepwise variable elimination.)

## Discussion

In this study we evaluated the outcome of cases through the assessment of abnormal physiological signs using the ABCD approach at a telephone triage. In this approach, the outcome of 31% of total cases was either moderate, severe, lethal, or dead. We transferred these cases to the hospital soon by ambulance without taking time to listen to the “patient’s other status indicators. The groupwise analysis found AD, BC, CD, ABD, and ABCD groups had a significantly larger proportion of the cases with severity, while the C and AB groups were significantly smaller than that in the total cases. Older age, male sex, and factors B and D were significantly associated with severity.

We already showed that 33% of cases, who were assigned to category red by Step 3 (symptom-specific protocols) in the #7119 telephone triage were moderate, severe, lethal, and dead, as in cases with severity according to the definition of this study [22]. Katayama et al. reported that 29.2% of patients transported by ambulance after telephone triage were hospitalized [23]. We share almost the same protocol of telephone triage and prehospital emergency ambulance system as their study in Japan. Patients with our definition of severity will be hospitalized at least (Table 1). In the present study, 31% of the cases were triaged using the ABCD approach (Step 2 in #7119) were the cases with severity and were hospitalized. These facts suggest that, at the telephone triage system in Japan, about 30% of cases, who are assigned to the red category by telephone triage and transported to hospital by ambulance, would be hospitalized at least based on their severity.

Wouters et al. reported that telephone triage nurses interpret the vocal—but not worded—elements in communication (paralanguage) such as tone of voice and shortness of breath and create a mental image to compensate for lack of visual information [24]. Croskerry pointed that two fundamental approaches to clinical reasoning have been recognized at a diagnosis. This dichotomy is now widely recognized as dual process theory, as System 1 which is automatic, fast and intuitive, and System 2, which is deliberate, reliable and analytical [25]. In the present study, triage nurses may assess patients with B and D to have abnormal paralanguage signs using tone of voice and shortness of breath, although they can’t see patients and can’t approach patient’s physical signs. It may indicate that triage nurses could pick up such severe patients by paralanguage signs with System 1 and could dispatch them by ambulance sooner. Further studies involving the analysis of the real records of each consultation are needed to revise the ABCD approach.

Haraldseide et al. reported that male sex was associated with a higher degree of urgent priority than female sex at the consultation, including by telephone and at primary healthcare centers. The urgent priority degree is a decision support tool used to determine response patterns and the degree of urgency at the consultation scene with the Norwegian Index of Emergency Medical Assistance. They discussed that consultation nurses generally perceive men as more urgent cases than women, partially because of symptom presentation [26]. In the present study, male sex was associated with severe outcomes, decided by physicians even after transport to hospital, in cases with red category triaged by telephone triage nurses. Cases of male sex, assigned to the severe category by telephone triage, may be associated with severe medical situation. However, our data does not contain enough information to analyze these sex differences. Therefore, future studies should be conducted based on nurses’ triage decisions to elaborate on sex differences.

At this study there were few cases at group AC, B, D and BD. I imagine some reasons for this phenomenon. First, patients in these categories may not consult telephone triage and may call an emergency number because its physiological abnormality may be obvious. Second, during the telephone interview, the triage nurses could not catch up with AC, B, D, or BD as severe signs enough to assign a patient to the category red. This may be partially because the data that triage nurses could obtain may be only the voice of the caller through the telephone, and they could not make a distinction between patients with severe cases in these groups. However, further studies are required to investigate this.

This study has some limitations. Unfortunately, we have not yet developed a standard to validate the acuity evaluated in the prehospital setting. Therefore, in order to check the validity of the “acuity,” we had to use a 5-category “severity” scale, including lethal, severe, moderate, mild, and dead upon admission in the ED categories. This is a limitation of this study. We need to discuss how to validate the outcomes of telephone triage referring to other criteria, such as the use of lifesaving interventions [16], guidelines for intensive care unit admission, discharge, and triage [27] or therapeutic intervention scoring system [28, 29]. This study did not evaluate inter-rater reliability and had possible misclassification due to the experience level of triage nurses. To solve this, we need to evaluate the response of at least two nurses to the recorded voice of patients..

## Conclusion

We concluded that at the #7119 telephone triage, we can pick up the severe cases soon without resorting to many questions by using the ABCD approach. These results can contribute to the prompt transportation of severe patients to hospitals by dispatch ambulance cars by the #7119 telephone triage. In the future, we need more investigation on why complaints involving combinations of factors B and D indicate severe cases using the ABCD approach in telephone triages.

## Data Availability

The excel data used to support the findings of this study may be released upon application to the #7119 handling committee of the Tokyo Medical Association who can be contacted at sakurai.atsushi@nihon-u.ac.jp.

## Abbreviations

MTS: Manchester Triage System
CTAS: Canadian Emergency Department Triage and Acuity Scale
ED: Emergency department
A: Airway
B: Breathing
C: Circulation
D: Dysfunction of the central nervous system
